# Empathy as related to gender, age, race and ethnicity, academic background and career interest: A nationwide study of osteopathic medical students in the United States

**DOI:** 10.1111/medu.14138

**Published:** 2020-04-02

**Authors:** Mohammadreza Hojat, Jennifer DeSantis, Stephen C. Shannon, Mark R. Speicher, Lynn Bragan, Leonard H. Calabrese

**Affiliations:** ^1^ Asano‐Gonnella Center for Research in Medical Education and Healthcare Sidney Kimmel Medical College at Thomas Jefferson University Philadelphia Pennsylvania USA; ^2^ American Association of Colleges of Osteopathic Medicine Bethesda Maryland USA; ^3^ Cleveland Clinic Lerner College of Medicine of Case Western Reserve University Cleveland Ohio USA

## Abstract

**Context:**

Research on associations between medical student empathy and demographics, academic background and career interest is limited, lacks representative samples and suffers from single institutional features. This study was designed to fill the gap by examining associations between empathy in patient care, and gender, age, race and ethnicity, academic background and career interest in nationwide, multi‐institutional samples of medical students in the United States and to provide more definitive answers regarding the aforementioned associations, with more confidence in the internal and external validity of the findings.

**Methods:**

Four nationwide samples participated in this study (n = 10 751). Samples 1, 2, 3 and 4 included 3616 first‐year, 2764 second‐year, 2413 third‐year and 1958 fourth‐year students who completed a web‐based survey at the end of the 2017‐2018 academic year. The survey included questions on demographics, academic background and career interest, the Jefferson Scale of Empathy, and the Infrequency Scale of the Zuckerman‐Kuhlman Personality Questionnaire to control for the effect of ‘good impression’ response bias.

**Results:**

Statistically significant and practically important associations were found between empathy scores and gender (in favour of women), race and ethnicity (in favour of African‐American and Hispanic/Latino/Spanish), academic background (in favour of ‘Social and Behavioural Sciences’ and ‘Arts and Humanities’ in Samples 1 and 2) and career interest (in favour of ‘People‐Oriented’ and ‘Psychiatry’ specialties).

**Conclusions:**

Special features of this study (eg, nationwide representative samples, use of a validated instrument for measuring empathy in patient care, statistical control for the effect of ‘good impression’ response bias, and consistency of findings in different samples from multiple institutions) provide more definitive answers to the issue of correlates of empathy in medical students and increase our confidence in the validity, reliability and generalisability of the results. Findings have implications for career counselling and targeting students who need more guidance to enhance their empathic orientation.

## Abstract 2

Using four nationwide samples of medical students in the US the authors explore correlates of empathy to explore strategies for career counselling and empathy development.

## 1 INTRODUCTION

Empathy is the heart of the art of patient care. Empathy has been described as the most frequently mentioned personal quality of the humanistic physician^1^ and a major element of professionalism in medicine.^2^ Cultivating empathy is listed amongst the goals of medical education, endorsed by professional medical organisations.^3^, ^4^, ^5^ Clinical empathy in patient care has been defined as a predominantly *cognitive* (as opposed to affective) attribute that involves an *understanding* of patient's experiences, concerns, pain and suffering, combined with a capacity to *communicate* this understanding and an *intention to help*.^6^ ^(p. 74)^

Empirical research shows that medical students’ empathy is positively associated with how faculty members rate their clinical competence,^7^ and physician's empathy has been found to predict positive clinical outcomes.^8^, ^9^ Significant correlations have also been reported between medical student's empathy and personality attributes conducive to relationship building (for a review see Hojat et al^10^ and Hojat et al^11^). Conversely, negative correlations have been found between empathy in medical and other health professions students and personal qualities detrimental to interpersonal relationships^. ^(for a review see Hojat et al^10^ and Hojat et al^11^). These findings suggest that empathic orientation can contribute both to the quality of interpersonal relations in general and to the outcomes of medical education and patient care in particular.

Although a number of studies have examined associations between empathy and gender amongst medical students,^10^, ^11^, ^12^, ^13^, ^14^, ^15^ few have researched empathy and career interest in medical students, and empirical research on medical students’ empathy in relation to age, race and ethnicity, and academic background is scarce. Almost all published studies on the aforementioned issues involve single‐institution research using small and non‐probabilistic accessible sampling designs that limit the internal and external validity of the findings. We designed this study to fill these gaps and shed light on associations between medical students’ empathy and gender, age, race and ethnicity, academic background and career interest with nationwide, multi‐institutional research, using large representative samples of medical students in the United States (US).

## 2 METHODS

### 2.1 Participants

Participants included a national sample of 10 751 (out of a total of 25 552) students in 41 of 48 campuses of osteopathic medical colleges in the USA (representing 85% of all osteopathic college campuses in the country). Students in all 4 years of medical school participated in the study, including 3616 (out of a total of 7197) first‐year students (Sample 1), 2764 (out of 6778) second‐year students (Sample 2), 2413 (out of 6683) third‐year students (Sample 3) and 1958 (out of 4894) fourth‐year students (Sample 4).

### 2.2 Study survey

We used a web‐based survey that consisted of questions about students’ gender, age, race and ethnicity, academic background and career interest, plus the following two scales:

#### 2.2.1 The Jefferson Scale of Empathy (S‐version)

This 20‐item instrument was developed by Hojat and colleagues^15^ for measuring clinical empathy in the context of patient care. Items are answered on a 7‐point Likert‐type scale (1 = strongly disagree; 7 = strongly agree). Ample evidence supports the psychometrics of the Jefferson Scale of Empathy (JSE) in samples of medical and other health professions students in the USA and abroad.^6^ ^(pp. 83‐128, 275‐286)^ The JSE has been translated into 56 languages, and used in more than 85 countries.^6^ Because of its worldwide use and extensive psychometric support, the JSE has been recognised as the most researched instrument in medical education research^16^ and the most frequently used instrument for measuring empathy in medical education.^17^ (A sample item: ‘Because people are different, it is difficult to see things from patients’ perspective.’)

Significant associations have been reported between medical students’ scores on the JSE and ratings of clinical competence in third‐year core clerkships given by medical school faculty members.^7^ Also, significant associations were observed between students’ JSE scores and ratings given by standardised patients in the objective structured clinical examination (OSCE) stations.^18^, ^19^ More importantly, significant associations have been reported between physicians’ scores on the JSE and tangible clinical outcomes in diabetic patients in the USA^8^ and abroad.^9^

Internal consistency reliability, determined by Cronbach's coefficient alpha, is mostly reported in the 0.70s and 0.80s,^6^ and stability of scores over time by test–retest reliability (in the 0.60s) has been reported in physicians,^20^ allopathic medical students^13^ and osteopathic medical students.^10^

#### 2.2.2 Measuring attempts to make ‘good impression’ responses

Respondents to self‐reported personality tests can manipulate their answers to produce good impressions. Such attempts to present a more socially acceptable version of ourselves are known as the ‘social desirability response set’^21^ and can confound research findings, leading to invalid conclusions.

Most of the JSE items are transparent; thus, respondents can produce ‘good impression’ answers. We used the ‘Infrequency’ Scale of the Zuckerman‐Kuhlman Personality Questionnaire (ZKPQ)^22^ to control for ‘good impression’ response bias. This 10‐item scale (true or false responses) was developed to identify subjects with invalid records (a sample item: ‘I never met a person that I didn't like’). According to the author of this scale, scores higher than 3 on this scale indicate questionable validity of the respondent's record.^22^ This scale has previously been used with medical students.^6^ ^(p. 127),^ ^23^

### 2.3 Procedure

The web‐based survey for this study evolved through several iterations and two pilot studies. The study was approved by the Institutional Review Board of Thomas Jefferson University and all other participating college campuses. We arranged to have one or two senior administrators or faculty‐level research coordinators from each participating college campus to serve as liaisons between the college and research teams at Jefferson and to schedule administration of the survey at their college campuses.

Prior to the survey administration, students in participating colleges were informed through campus announcements and email messages about the project and the importance of their participation. The survey was administered at the end of the 2017‐2018 academic year. With the exception of a voluntary option to enter the respondent's email address for receiving feedback, no personal identification information was solicited. All individual data were treated with strict confidentiality.

### 2.4 Statistical analyses

We used Pearson correlations to examine associations between JSE scores and age. Also, we used analysis of covariance in which group classification was the independent variable, the JSE score was the dependent variable and the score on the ‘Infrequency’ scale of the ZKPQ^22^ served as a covariate. We calculated effect sizes (mean differences in terms of standard deviation [SD] unit) for the statistically significant differences to determine the practical (clinical) importance of the statistically significant findings.^24^, ^25^ Effect sizes of 0.20 or less were considered negligible, thus practically unimportant.^24^, ^25^ Mean scores of the JSE were adjusted using the score of the ‘Infrequency’ scale of the ZKPQ as covariate, to control for the effect of ‘good impression’ response bias (less than 3% of respondents in each study sample scored above the cut‐off point of 3 on this scale).

## 3 RESULTS

### 3.1 Response rates, sample sizes and samples representativeness

Response rates varied for the different study samples. For example, response rates were 50% (3616/7197), 41% (2764/6778), 36% (2413/6683) and 40% (1958/4894) for Samples 1, 2, 3 and 4, respectively. To examine representativeness of the samples, we compared the study samples with their respective populations and found the samples closely resembled their respective populations with regard to gender, age and race and ethnicity. In other words, the differences between usable samples and their respective populations were not practically important on the aforementioned demographic variables (based on negligible effect sizes < 0.20). Population data were obtained from the American Association of Colleges of Osteopathic Medicine (AACOM) (detailed findings presented elsewhere).^11^

### 3.2 Gender

Women constituted 49% (*n* = 5271) of the total participants. Table 1 shows their composition in each sample: Sample 1, 48% (*n* = 1738); Sample 2, 50% (*n* = 1383); Sample 3, 49% (*n* = 1180); and Sample 4, 50% (*n* = 970). Mean scores and SDs on the JSE for each sample and total participants by gender, and summary results of statistical analyses are also presented in Table 1. In all four samples, women consistently obtained higher JSE mean scores than men. For the total participants, the mean score for men was 111.82 (SD = 13.30) and for women it was 116.96 (SD = 10.82); the difference was statistically significant (*F*_(1,10 619) _= 472.68, *P* < .01) and practically important (effect size = 0.42). A similar pattern of findings was observed in each of the study samples.

**Table 1:** Means^a^ and standard deviations of scores on the Jefferson scale of empathy for men and women^b^ in national samples from 41 campuses of United States Colleges of Osteopathic Medicine

Gender	Sample 1^c^		Sample 2^c^		Sample 3^c^		Sample 4^c^		Total	
	N (%)	M (SD)	N (%)	M (SD)	N (%)	M (SD)	N (%)	M (SD)	N (%)	M (SD)
Men	1839 (51)	112.43 (13.14)	1348 (49)	112.34 (12.63)	1198 (50)	111.12 (13.58)	966 (50)	110.74 (14.04)	5351 (50)	111.82 (13.30)
Women	1738 (48)	118.03 (10.60)	1383 (50)	117.19 (10.60)	1180 (49)	116.09 (11.06)	970 (50)	115.79 (11.07)	5271 (49)	116.96 (10.82)
Other^d^	39 (1)		33 (1)		35 (1)		22 (1)		129 (1)	
Adjusted *F*‐ratio	*F*_(1,3574)_ = 192.65**		*F*_(1,2728)_ = 116.19**		*F*_(1,2375)_ = 96.32**		*F*_(1,1933)_ = 76.87**		*F*_(1,10 619)_ = 472.68**	

Note

Group differences: in all analyses, women scored significantly higher than men.

M, mean; SD, standard deviation.

^a^ Mean scores were adjusted by using analysis of covariance to control for the effect of ‘good impression’ response bias. Scores of the Infrequency Scale of the Zuckerman–Kuhlman Personality Questionnaire served as covariate.

^b^ Respondents who did not report their gender or who were included in the ‘other’ category were excluded from the statistical analysis.

^c^ Samples 1‐4 included students in the first, second, third and fourth years of medical school who completed the study survey at the end of the 2017‐2018 academic year.

^d^ Included those who did not report their gender and those who reported: transgender male; transgender female; gender variant or non‐conforming; not listed, or decline to answer.

** *P* < .01.

### 3.3 Age

The mean age in Sample 1 was 25.5 years (SD = 3.2), in Sample 2 it was 26.3 years (SD = 3.2), in Sample 3 it was 27.4 years (SD = 3.4) and in Sample 4 it was 28.6 years (SD = 3.5). We calculated Pearson correlation coefficients between scores of the JSE and students’ ages in the four study samples and total participants. The magnitudes of the correlation coefficients were small (ranging from 0.00 to 0.10). Correlations of these magnitudes are negligible and practically unimportant.

### 3.4 Race and Ethnicity

The majority of respondents in each of the study samples were White/Caucasian (65% of the total participants, *n* = 7022), followed by Asian (21% of the total participants, *n* = 2304), Hispanic/Latino/Spanish (5% of the total participants, *n* = 485) and African‐American (3% of the total participants, *n* = 324). (Table 2). In each of the samples, 1% or fewer identified as American Indian/Alaskan or Hawaiian/Pacific Islander. The JSE mean scores, SDs and summary results of statistical analyses are reported in Table 2.

**Table 2:** Means^a^ and standard deviations of scores on the Jefferson scale of empathy by race and ethnicity^b^ in national samples from 41 campuses of United States Colleges of Osteopathic Medicine

Race and ethnicity category	Sample 1^c^		Sample 2^c^		Sample 3^c^		Sample 4^c^		Total	
	N (%)	M (SD)	N (%)	M (SD)	N (%)	M (SD)	N (%)	M (SD)	N (%)	M (SD)
White/Caucasian	2283 (63)	115.34 (12.08)	1805 (65)	114.76 (11.95)	1609 (67)	113.66 (12.31)	1325 (68)	113.38 (12.82)	7022 (65)	114.44 (12.27)
African‐American	107 (3)	117.08 (11.50)	83 (3)	118.66 (12.26)	78 (3)	117.63 (11.39)	56 (3)	115.19 (13.42)	324 (3)	117.28 (12.01)
Hispanic/Latino/Spanish	207 (6)	116.56 (11.74)	114 (4)	115.87 (10.29)	100 (4)	113.84 (13.63)	64 (3)	116.17 (11.92)	485 (5)	115.77 (11.88)
Asian	842 (23)	114.32 (12.88)	607 (22)	114.47 (11.82)	470 (19)	112.69 (13.74)	385 (20)	112.07 (12.74)	2304 (21)	113.66 (12.80)
Other^d^	177 (5)		155 (6)		156 (6)		128 (6)		616 (6)	
Adjusted *F*‐ratio	*F*_(3,3434) _= 3.25*		*F*_(3,2604)_ = 3.40*		*F*_(3,2252)_ = 3.57*		*F*_(3,1825)_ = 2.72*		*F*_(3,10 130)_ = 10.78**	

Note

Group differences: for Sample 1: Asian < all ethnic groups; for Sample 2: African‐American > Asian, African‐American > White/Caucasian; for Sample 3: African‐American > all ethnic groups; for Sample 4: Hispanic/Latino/Spanish > Asian. In the total sample (samples 1–4 combined), all pairwise comparisons were statistically significant with the exception of Hispanic/Latino/Spanish compared to African‐American, which showed no statistical difference in adjusted mean scores.

M, mean; SD, standard deviation.

^a^ Mean scores were adjusted by using analysis of covariance to control for the effect of ‘good impression’ response bias. Scores of the Infrequency Scale of the Zuckerman–Kuhlman Personality Questionnaire served as covariate.

^b^ Respondents who did not report their ethnicity or who were included in the ‘Other’ category were excluded from statistical analysis.

^c^ Samples 1–4 included students in the first, second, third and fourth years of medical school who completed the study survey at the end of the 2017‐2018 academic year.

^d^ Includes students who did not report their race and ethnicity and those who identified as American Indian/Alaskan or Hawaiian/Pacific Islander

* *P* < .05.

** *P* < .01.

The highest JSE mean score for the total participants was obtained by African‐American students (mean [*M*] = 117.28, SD = 12.01), and the lowest by Asians (*M* = 113.66, SD = 12.80; effect size was 0.29). A similar pattern of differences was observed in all of the study samples, with the exception of Sample 4 in which the Hispanic/Latino/Spanish group obtained a significantly higher mean score (*M* = 116.17, SD = 11.92), closely followed by the African‐American group (*M* = 115.19, SD = 13.42). Asians obtained the lowest empathy mean score in this sample (*M* = 112.07, SD = 12.74). Significant post hoc pairwise mean differences are reported in the footnotes of the tables.

### 3.5 Academic background

Respondents could choose from a list of 56 undergraduate majors, in alphabetical order, as well as the options of ‘Other’ or ‘No Major’ (an undergraduate college degree is required for application to US medical schools, which often consists of 4 years of medical education to earn a medical degree). For the purpose of statistical analysis, we grouped the undergraduate majors into the following broad categories: ‘Biological Sciences;’ ‘Chemical and Physical Sciences;’ ‘Social and Behavioural Sciences;’ ‘Arts and Humanities,’ and ‘Other.’

The majority of students majored in ‘Biological Sciences’ (60% of the total participants, *n* = 6423) and the fewest majored in ‘Arts and Humanities’ (4% of the total participants, *n *= 401). The pattern of distribution of undergraduate majors was similar in the four study samples (Table 3). Means and SDs of the JSE scores by academic background and summary results of statistical analyses are presented in Table 3.

**Table 3:** Means^a^ and standard deviations of scores on the Jefferson scale of empathy by undergraduate major^b^ in national samples from 41 campuses of United States Colleges of Osteopathic Medicine

Undergraduate major	Sample 1^c^		Sample 2^c^		Sample 3^c^		Sample 4		Total	
	N (%)	M (SD)	N (%)	M (SD)	N (%)	M (SD)	N (%)	M (SD)	N (%)	M (SD)
Biological Sciences	2177 (60)	115.04 (12.52)	1617 (58)	114.40 (12.02)	1488 (62)	113.43 (13.18)	1141 (58)	113.13 (12.68)	6423 (60)	114.17 (12.60)
Chemical and Physical Sciences	545 (15)	114.02 (12.98)	466 (17)	113.47 (12.86)	345 (14)	112.48 (13.47)	312 (16)	112.43 (14.41)	1668 (16)	113.23 (13.34)
Social and Behavioural Sciences	246 (7)	116.29 (12.00)	213 (8)	116.56 (12.39)	163 (7)	114.59 (12.13)	153 (8)	115.17 (12.59)	775 (7)	115.80 (12.25)
Arts and Humanities	117 (3)	117.10 (11.62)	101 (4)	116.68 (11.27)	109 (5)	114.25 (12.57)	74 (4)	113.31 (13.63)	401 (4)	115.53 (12.24)
Other^d^	531 (15)		367 (13)		308 (12)		278 (14)		1484 (14)	
Adjusted *F*‐ratio	*F*_(3,3080)_ = 3.09*		*F*_(3,2392)_ = 4.29**		*F*_(3,2100)_ = 1.20		*F*_(3,1675)_ = 1.57		*F*_(3,9262)_ = 8.95**	

Note

Group differences: for Sample 1*:* Arts and Humanities > Chemical and Physical Sciences, Social and Behavioural Sciences > Chemical and Physical Sciences; for Sample 2: Arts and Humanities > Chemical and Physical Sciences, Social and Behavioural Sciences > Biological Sciences, Social and Behavioural Sciences > Chemical and Physical Sciences. No statistically significant differences were observed in Samples 3 and 4. In the total sample (Samples 1–4 combined), each pairwise comparison was statistically significant, with the exception of Arts and Humanities compared to Social and Behavioural Sciences, which showed no statistical difference in adjusted mean scores.

Abbreviations: M, mean; SD, standard deviation.

^a^ Mean scores were adjusted by using analysis of covariance to control for the effect of ‘good impression’ response bias. Scores of the Infrequency Scale of the Zuckerman–Kuhlman Personality Questionnaire served as covariate.

^b^ Respondents who did not report their undergraduate major or who reported majors in the ‘other’ category were excluded from statistical analysis.

^c^ Samples 1–4 included students in the first, second, third and fourth years of medical school who completed the study survey at the end of the 2017‐2018 academic year.

^d^ Includes those who did not report their undergraduate major, and those who reported the following majors: Double Major in Science and Non‐Science; General Studies; Honors Program; Interdisciplinary; Pre‐Med; Other, and No Major.

* *P* < .05.

** *P* < .01.

The highest mean scores for empathy of the total participants were obtained by students who majored in ‘Social and Behavioural Sciences’ (*M* = 115.80, SD = 12.25) and ‘Arts and Humanities’ (*M* = 115.53, SD = 12.24), and the lowest mean score was obtained by those with a background in ‘Chemical and Physical Sciences’ (*M* = 113.23, SD = 13.34). The differences were statistically significant (*F*_(3,9262) _= 8.95, *P* < .01) but practically unimportant (effect size between the extreme groups < 0.20). A similar pattern of findings was observed in the four study samples. However, differences in the JSE scores by academic background were not statistically significant for Samples 3 and 4, but were statistically significant and practically important in Sample 1 (*F*_(3,3080)_ = 3.09, *P* < .05, effect size between extreme groups = 0.24) and in Sample 2 (*F*_(3,2392)_ = 4.29, *P* < .01, effect size between extreme groups = 0.25).

### 3.6 Career interest

A list of the 23 specialties most frequently pursued by graduates of colleges of osteopathic medicine was included in the survey, as well as options of ‘Other’ and ‘Undecided.’ Respondents were asked to indicate the specialty they planned to pursue after medical school.

We classified the specialties into the following broad categories: ‘People‐Oriented‘ (including Family Medicine, Internal Medicine, Obstetrics and Gynaecology, and Paediatrics); ‘Technology and Procedure‐Oriented’ (including Anaesthesiology, Dermatology, Neurological Surgery, Ophthalmology, Orthoapedic Surgery, Otolaryngology and Facial Plastic Surgery, Pathology, Plastic Surgery, Radiology, and Surgery), and ‘Other’ specialties (including specialties chosen by fewer than 20 students). We retained ‘Psychiatry’ in its own category because in previous research, psychiatrists obtained the highest scores on the JSE^20^ and we were interested to ascertain if that was also the case with osteopathic medical students.

Grouping specialties into broad categories of ‘People‐Oriented’ and ‘Technology and Procedure‐Oriented’ has been used in medical education research.^6^, ^12^, ^20^, ^26^ Specialties that require frequent and continuous encounters with patients and preventive care consultations are grouped in the ‘People‐Oriented” specialties and specialties that require more technical and procedural skills are grouped in the ‘Technical and Procedure‐Oriented’ specialties.

In this study, ‘People‐Oriented’ was the most frequently chosen category in each study sample and was selected by 45% (*n* = 4867) of the total participants. The proportion expressing an interest in pursuing a ‘People‐Oriented’ specialty (mostly a primary care specialty) increased as students progressed through medical school (from 35% in Sample 1, *n* = 1257, to 63% in Sample 4, *n* = 1231) (Table 4).

**Table 4:** Means^a^ and standard deviations of scores on the Jefferson Scale of Empathy by specialty career plan^b^ in national samples from 41 campuses of United States Colleges of Osteopathic Medicine

Specialty	Sample 1^c^		Sample 2^c^		Sample 3^c^		Sample 4^c^		Total	
	N (%)	M (SD)	N (%)	M (SD)	N (%)	M (SD)	N (%)	M (SD)	N (%)	M (SD)
People‐Oriented^d^	1257 (35)	116.73 (11.65)	1102 (40)	116.41 (10.89)	1277 (53)	114.56 (12.45)	1231 (63)	114.17 (12.29)	4867 (45)	115.43 (11.92)
Technology and Procedure‐Oriented^e^	705 (19)	112.28 (13.35)	427 (15)	111.95 (13.66)	495 (20)	110.13 (14.41)	273 (14)	110.53 (15.40)	1900 (18)	111.41 (14.03)
Psychiatry	72 (2)	117.58 (12.81)	88 (3)	117.27 (13.54)	134 (6)	116.64 (12.10)	102 (5)	116.21 (11.11)	396 (4)	116.93 (12.28)
Other specialties^f^	740 (20)		501 (18)		441 (18)		342 (17)		2024 (19)	
Undecided	842 (23)		646 (23)		66 (3)		10 (<1)		1564 (15)	
Adjusted *F*‐ratio	*F*_(2,2030)_ = 31.00**		*F*_(2,1613)_ = 23.03**		*F*_(2,1902)_ = 25.36**		*F*_(2,1602)_ = 11.29**		*F*_(2,7159)_ = 78.88**	

Note

Group differences: for all samples: Technology and Procedure‐Oriented < Psychiatry, Technology and Procedure‐Oriented < People‐Oriented. In the total sample (samples 1–4 combined) the pairwise comparison of Psychiatry > People‐Oriented was also statistically significant.

Abbreviations: M, mean; SD, standard deviation.

^a^ Mean scores were adjusted by using analysis of covariance to control for the effect of ‘good impression‘ response bias. Scores of the Infrequency Scale of the Zuckerman–Kuhlman Personality Questionnaire served as covariate.

^b^ Respondents who did not report their specialty plan, those who were undecided or those who reported specialties in the ‘other’ category were excluded from statistical analysis.

^c^ Samples 1–4 included students in the first, second, third and fourth years of medical school who completed the study survey at the end of the 2017‐2018 academic year.

^d^ People‐oriented specialties included: Family Medicine; Internal Medicine; Obstetrics and Gynaecology, and Paediatrics

^e^ Technology and Procedure‐Oriented specialties included: Anaesthesiology; Dermatology; Neurological Surgery; Ophthalmology; Orthoapedic Surgery; Otolaryngology and Facial Plastic Surgery; Pathology; Plastic Surgery; Radiology, and Surgery.

^f^ Includes those who did not report their specialty plan, those who selected ‘Other’ specialty or those who selected specialties that were chosen by <20 students.

** *P* < .01.

Students interested in ‘People‐Oriented,’ ‘Technology/Procedure‐Oriented’ and ‘Psychiatry’ specialties were compared on their empathy scores. Means and SDs of the JSE scores and summary results of statistical analyses are reported in Table 4. Those of the total participants who were interested in pursuing ‘Psychiatry’ (*M* = 116.93, SD = 12.28) and ‘People‐Oriented’ (*M* = 115.43, SD = 11.92) specialties obtained higher mean empathy scores than those interested in ‘Technology and Procedure‐Oriented’ (*M* = 111.41, SD = 14.03) specialties (*F*_(2,7159)_ = 78.88, *P* < .01, effect sizes = 0.40 and 0.32, respectively). The pattern of findings was similar in the four study samples.

## 4 DISCUSSION

Our results confirm some of the previous findings about associations between empathy, gender and career interest and provide new insights into associations between empathy, race and ethnicity and academic background. Our finding of higher empathy scores amongst women aligns with most of those reported in allopathic^12^, ^13^ and osteopathic medical students.^14^, ^15^, ^27^ The gender difference in empathy has often been attributed to social learning and cultural factors.^6^ However, evidence regarding gender‐specific behaviours observed in infants and toddlers (eg, infant's reactive crying)^28^ suggests that women's empathic inclination may have hard‐wired roots, in addition to reflecting social learning and cultural factors.

Empirical research on empathy and age is scarce in medical students. Our findings of no substantial correlation between JSE scores and age agree with a few studies in health professions students.^6^ ^(p159)^ It may be speculated that the negligible correlation between JSE and age in health professions students could be an artifact of the ‘restriction of range’ phenomenon in students’ ages, which does not allow the corresponding correlation to capture the full range of the relationship. More research is needed to confirm this speculation.

Empirical research on empathy and race and ethnicity in medical students is scarce. One reason is that such studies undertaken in a single institution often lack a sufficient number of available students in the under‐represented race and ethnic groups to allow meaningful statistical analyses. Our findings that African‐American as well as Hispanic/Latino/Spanish students obtained the highest mean empathy scores are interesting and call for further research to explore underlying reasons. A study with nursing students^29^ found no significant association between race and ethnicity and scores on the JSE. However, consistent with our findings, in a multi‐institutional study with allopathic medical students, African‐American students obtained significantly higher JSE scores than White/Caucasian and Asian/Pacific Islander students.^19^

The higher JSE scores in the under‐represented African‐American and Hispanic/Latino/Spanish minority groups may be explained by the notion of the “wounded healer effect”^6^ ^(p140),^ ^30^ , which describes that those who have experienced suffering can better understand the suffering of others by sharing common experiences. This effect suggests that those who have experienced discrimination and social injustice may be more sensitive to the suffering of others and develop more empathic understanding of others who are in need of help.

Empirical research on empathy and students’ academic backgrounds is also scarce. This is the first large‐scale study to examine associations between empathy and undergraduate majors in medical students. Our findings do not agree with those reported in nursing students^29^, ^31^ in which no significant association was observed between academic background (in humanities, sciences and business) and the JSE scores. Similarly, Smolarz^32^ did not find a significant difference in JSE scores amongst first‐year medical students who majored in science and non‐science disciplines. Interestingly, in the present study, differences in empathy by undergraduate majors were observed in the pre‐clinical phase of medical school (Years 1 and 2 of medical school), but these differences faded in the clinical phase (Years 3 and 4). This change of findings suggests that the effects of academic background on empathic orientation towards patient care might last only during the early years of medical school education. More empirical research is needed to confirm this speculation.

Consistent with our findings, several studies have reported significant differences in empathy scores in allopathic medical students who expressed an interest in a ‘People‐Oriented’ specialty and those who expressed an interest in a ‘Technology and Procedure‐Oriented’ specialty.^12^ This pattern of findings has also been reported amongst practising physicians.^20^ In her doctoral dissertation, Bailey^33^ reported that medical students who planned to pursue a career in specialties requiring extensive and prolonged encounters with patients received significantly higher empathy scores than their counterparts who planned to pursue procedure‐oriented specialties. Previous studies on specialty interest and empathy in osteopathy medical students reported mixed results. One study reported that students who were planning to pursue ‘People‐Oriented’ specialties scored higher on the JSE than their peers who were planning to pursue ‘Technology and Procedure‐Oriented’ specialties.^34^ However, another study with osteopathy medical students did not find such a relationship.^27^

There are some differences between allopathic and osteopathic medical education philosophies. For example, in osteopathic medical education, a greater emphasis is placed on provision of holistic care, hands‐on approaches to diagnosis and treatment, and integrative patient‐centred care.^35^, ^36^ Thus, it is important to examine similarities and differences in research findings on empathy between allopathic and osteopathic medical students. Findings of this study regarding associations between empathy, gender and specialty interest are generally consistent with those in allopathic medical schools. We need comparable data on ethnicity and academic background to explore similarities and differences.

### 4.1 Implications

Findings of associations between empathy and gender, ethnicity, academic background and specialty interest have implications for identifying medical students who may need additional help to enhance and sustain their empathic orientation towards patient care. For example, empirical evidence suggests that empathy tends to decline in both allopathic^6^, ^37^ and osteopathic^14^, ^38^ medical students. Given the findings of this study and taking into consideration the limited resources for offering remedial educational programmes, it is important to identify students who need more than others to benefit from such remedies, such as male students, White students and those with academic backgrounds in majors other than the ‘Humanities and Arts,’ and interest in ‘Technology and Procedure‐Oriented’ specialties. Empirical findings that suggest empathy can be enhanced and sustained in physicians‐in‐training^39^ by exposing them to special goal‐directed programmes provide additional support for the aforementioned implications. Also, the findings have implications for career counselling and guiding students with different empathy scores in choosing ‘People‐Oriented’ or ‘Technology and Procedure‐Oriented’ specialties.

### 4.2 Limitations and strengths

A limitation of this study is the lower than 50% response rates in Samples 2‐4. However, evidence supporting the representativeness of the study samples with regard to age, gender and race and ethnicity mitigates this shortcoming. Also, we do not know if the empathic orientation of non‐respondents would be similar to their respondent counterparts. Despite these limitations, this study benefits from several strengths, including: (a) four nationwide samples from multiple institutions; (b) use of a well‐established empathy‐measuring instrument specifically developed for administration to medical students, with face and content validities and strong psychometric support in both allopathic and osteopathic medical students; (c) statistical control for ‘good impression’ response bias, and (d) consistency of results across different study samples. These features increase our confidence about the internal validity (true relationships amongst variables) and external validity (generalisation) of the results, thus providing more definitive answers to the issues addressed in this study.

## 5 CONCLUSIONS

This nationwide study of empathy in osteopathic medical students offers the most definitive insights to date into associations between empathic orientation in patient care and gender, race and ethnicity, academic background and career interest amongst osteopathic medical students. Our results have implications for medical students’ career counselling and can also help medical schools monitor and target those who need more guidance to improve and sustain their empathic orientation towards patient care.

## AUTHOR CONTRIBUTIONS

MH, SCS and LHC contributed to the inception of this research and its design. JDS, MRS and LB contributed to data collection and data management. MH and JDS contributed to statistical analyses of data. MH, JDS, SCS and LHC contributed to interpretation of findings. All authors (MH, JDS, SCS, MRS, LB, and LHC) contributed to the writing of the manuscript and approved the final manuscript.

## CONFLICTS OF INTEREST

The authors declare that they do not have a conflict of interest.

## ETHICAL APPROVAL

This study was approved by the Institutional Review Boards of Thomas Jefferson University and all participating colleges.
